# Impact of suppression of tumorigenicity 14 (ST14)/serine protease 14 (Prss14) expression analysis on the prognosis and management of estrogen receptor negative breast cancer

**DOI:** 10.18632/oncotarget.9155

**Published:** 2016-05-04

**Authors:** Sauryang Kim, Jae Woong Yang, Chungho Kim, Moon Gyo Kim

**Affiliations:** ^1^ Inha University, Department of Biological Sciences, Incheon, Republic of Korea; ^2^ Department of Life Sciences, Korea University, Seoul, Republic of Korea; ^3^ Convergent Research Institute for Metabolism and Immunoregulation, Incheon, Republic of Korea

**Keywords:** ST14, Prss14, epithin, breast cancer, epithelial mesenchymal transition (EMT)

## Abstract

To elucidate the role of a type II transmembrane serine protease, ST14/Prss14, during breast cancer progression, we utilized publically accessible databases including TCGA, GEO, NCI-60, and CCLE. Survival of breast cancer patients with high ST14/Prss14 expression is significantly poor in estrogen receptor (ER) negative populations regardless of the ratios of ST14/Prss14 to its inhibitors, SPINT1 or SPINT2. In a clustering of 1085 selected EMT signature genes, ST14/Prss14 is located in the same cluster with CDH3, and closer to post-EMT markers, CDH2, VIM, and FN1 than to the pre-EMT marker, CDH1. Coexpression analyses of known ST14/Prss14 substrates and transcription factors revealed context dependent action. In cell lines, paradoxically, ST14/Prss14 expression is higher in the ER positive group and located closer to CDH1 in clustering. This apparent contradiction is not likely due to ST14/Prss14 expression in a cancer microenvironment, nor due to negative regulation by ER. Genes consistently coexpressed with ST14/Prss14 include transcription factors, ELF5, GRHL1, VGLL1, suggesting currently unknown mechanisms for regulation. Here, we report that ST14/Prss14 is an emerging therapeutic target for breast cancer where HER2 is not applicable. In addition we suggest that careful conclusions should be drawn not exclusively from the cell line studies for target development.

## INTRODUCTION

Type II transmembrane serine proteases are a family of proteases that play physiological roles including embryo development and maintenance of homeostasis [1, 2]. A representative member is protease serine 14 (Prss14), originally called Epithin when it was cloned from thymic epithelium [3]. It has been also called as matriptase [4], membrane type serine protease 1 (MT-SP1) [5], and suppression of tumorigenicity 14 (ST14) [6]. We will use ST14/Prss14 as the name hereafter.

ST14/Prss14 is a protease normally on the cell surface with processing at the Gly 149 residue during biogenesis [7]. The N terminal fragment containing cytoplasmic, transmembrane domains, and a short ectodomain is linked to the rest of the C-terminal fragment containing CUB, LDLR, and a protease domain. Protease activity of ST14/Prss14 requires cleavage at Arg 614, presumably by autocatalysis (reviewed in [8–10]). Upon TGFβ induction, the ectodomain is further processed by the TNFα converting enzyme (ADAM17) to be shed from the membrane [11]. Phorbol ester can also induce shedding by regulating ST14/Prss14 binding to filamin, a cytoplasmic actin binding protein [12]. An isoform of ST14/Prss14 missing the LDLRA4 domain is defective in shedding [7]. Shed protein recovered from a thymoma cell line and transfected cells behaves as a proangiogenic factor [13]. In addition, we showed that ST14/Prss14 is necessary and sufficient for epithelial mesenchymal transition (EMT) [14]. It also plays an important role in transendothelial migration of epithelial cancer cells [15] and activated macrophage [16].

There has been increasing attention on the normal and pathological cellular function of ST14/Prss14 (summarized in [8, 17, 18]). Its biological functions largely depend on the diverse substrates, such as extracellular matrix proteins [19], growth factors including hepatocyte growth factor (HGF) [20], epidermal growth factor receptor (EGFR) [21], adhesion molecules including Mac25 [22] and Tie2 [15], proteases including urokinase plasminogen activator (uPA) [20], prostasin (PRSS8) [23], matrix metalloprotease 3 (MMP3) [24], and more. Two protease inhibitors, HGF activator inhibitor type 1 and 2 (HAI-1 and HAI-2, gene names, SPINT1 and SPINT2, respectively), are known to bind to the ST14/Prss14 protease domain. Serum factors, steroid hormones, acidic pH and changes in ionic strength in the cellular microenvironments can release the inhibitors from the protease and activate it (reviewed in [9, 10, 18, 25]). It is suggested that ST14/Prss14 may be the most upstream of the protease cascade and can control multiple downstream functions.

Physiological functions of ST14/Prss14 are revealed by genetically modified mouse models [17, 26–29] and genetic mutations observed in human populations with a rare skin phenotype [30]. ST14/Prss14 primarily functions in epithelial tissues including skin and thymus. When regulated by the inhibitors or by overexpression, most phenotypes appear as forms of malignant tumors in epithelial tissues [31]. For example, skin-specific overexpression of ST14/Prss14 under Keratin 5 promoter in mouse induced spontaneous skin hyperplasia advanced further into squamous cell carcinoma [26]. Recently, its role in accumulation of inflammation was reported [28]. By using multi-transgenic crosses to manipulate expression of ST14/Prss14 and its inhibitor SPINT2, Thomas Bugge's group showed that ST14/Prss14 can initiate tumorigenesis by causing tumor-associated inflammation.

Genetic profiling, such as a gene expression array analysis, revealed that overexpression of ST14/Prss14 was frequently found in advanced epithelial cancers [32–34]. However, as the name suggests, ‘suppression’ of tumors by ST14/Prss14 was claimed based on the observation that the expression is reduced in colon cancer [6]. Indeed, a study using systemic expression profiling and immunohistochemistry (IHC) showed that its expression is lower in advanced ovarian cancers [35]. Thus, it is confusing to determine whether ST14/Prss14 has pro- or anti- carcinogenic activity. It appears that sample sizes, tissue types, and context with microenvironments influence the outcome of the results. Furthermore, variations in expression of its cognate inhibitors, SPINT1 and SPINT2, seem to add another level of complexity [36, 37]. Therefore, careful detailed analyses on expression profiling are necessary to clarify the role of the ST14/Prss14 in specific cases.

Systemic expression profiling using cancer patients' tissue samples brought exciting new windows for selecting prognosis markers and therapeutic targets. For the breast cancers especially, genetic profiling analysis opened a new way to classify cancer types. For a good example, breast cancers can be divided into four different types through the Cancer Genome Atlas (TCGA) study, based on the expression of three important marker genes, estrogen receptor (ER), human EGF receptor 2 (HER-2) and progesterone receptor (PR) [38, 39]. Four breast cancer subtypes are luminal A (ER^+^, PR^+/−^, HER-2^−^), luminal B (ER^+^, PR^+/−^, HER-2^+^), HER-2-enrich (ER^−^, PR^−^, HER-2^+^), and basal-like (ER^−^, PR^−^, HER-2^−^), triple negative (TN).

Another important parameter derived from the expression profiling is the signature genes of epithelial-to-mesenchymal transition (EMT). EMT is an event that involves massive gene expression changes, resulting in switches from one type of more epithelial into the other type of more mesenchymal shape and characteristics (reviews in [40, 41]). After EMT, cancer cells acquire abilities of penetrating basement membranes and blood vessels, and of moving to distant sites. As a result, another focus of cancer cells can settle and grow as secondary tumors. Data on EMT signature genes are being accumulated from studies using model cancer cell lines in defined conditions [42–44]. For example, during EMT progression, E-cadherin (CDH1) expression decreases, vimentin (VIM) expression increases transiently, while N-cadherin (CDH2) and fibronectin (FN1) expression increase [45]. However, detail mechanisms remain as questions at this time point as to how EMT proceeds and which transcription factors govern the particular signal pathways, making it difficult to connect the whole picture.

In this study, we attempted to clarify the role of ST14/Prss14 in breast cancer progression and patients' survival using public databases of gene expression profiles. Through the analyses, we suggest that ST14/Prss14 is an excellent prognosis marker and therapeutic target for ER^−^/TN breast cancers. We also propose yet-to be-identified modes of action for ST14/Prss14 during EMT.

## RESULTS

### ST14/Prss14 is a superior prognosis marker for ER negative breast cancer

**Figure 1:**
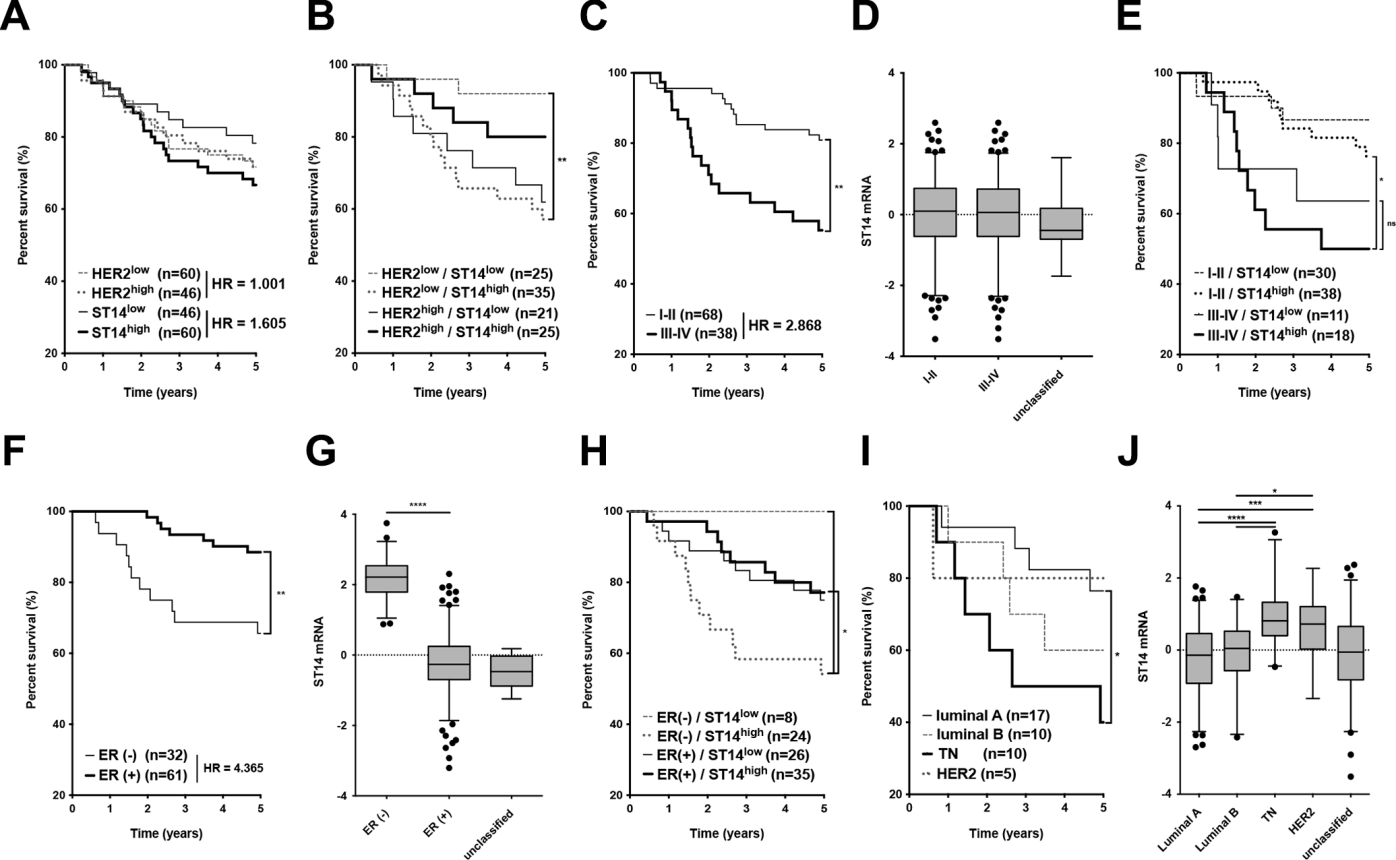
Analysis of survival and ST14/Prss14 expression in the TCGA BRCA dataset (**A**) Kaplan-Meier survival curves for the high and the low expression groups of ST14/PRSS14 and HER2. Each group was separated by the mean expressions of ST14/Prss14 and of HER2. The Hazard Ratio (HR) is shown in the figure. (**B**) Four groups were divided first by the highs and the lows of HER2 expression and next by the highs and the lows of ST14/Prss14 expression. (**C**) An analysis of survival by cancer stages I–II and III–IV. The Hazard Ratio (HR) is shown in the figure. (**D**) Box plots of ST14/Prss14 expression by cancer stages I–II and III–IV. (**E**) Kaplan-Meier survival curves of four groups first by cancer stages I–II and III–IV and second by the expressions of ST14/Prss14. (**F**) Survival by ER expression status. Kaplan-Meier survival curves of two groups by ER status determined by IHC. The Hazard Ratio (HR) is shown in the figure. (**G**) Box plots of ST14/Prss14 expression by ER status. Mean Difference of ER^−^ vs. ER^+^, 2.440. (**H**) Kaplan-Meier survival curves of four groups separating first by ER status and next by ST14/Prss14 expression. (**I**) Kaplan-Meier survival curves of four groups by breast cancer subtypes. (**J**) Box plots of ST14/Prss14 expression by breast cancer subtypes. Mean Difference, TN vs. luminal A: 1.075, TN vs. luminal B: 0.935, TN vs. HER2: 0.240. In box plots, the median was plotted as a line in the middle of the gray box. The whiskers were drawn down to the 2.5 percentiles and up to the 97.5 percentiles. Points below and above the whiskers were outlier dots. A one-way ANOVA was calculated between groups and *P* values were determined by Sidak's multiple comparisons test. *P < 0.05, **P < 0.01, ***P < 0.001, ****P < 0.0001.

Previously, we reported that ST14/Prss14 can enhance metastasis in a mouse breast cancer model [15]. To investigate whether the conclusion drawn from the mouse study is valid in human breast cancer patient population, we searched ST14/Prsss14 expression by using a well-documented TCGA BRCA database. We first examined survival rates of breast carcinoma patients grouped according to ST14/Prss14 expression levels with assignment of “high” and “low” (relatively higher or lower than the average of the whole data points, respectively) and calculated hazard ratio (HR) for each group by using the Mantel-Haenszel method. The ST14/Prss14^high^ group show poorer survival than the ST14/Prss14^low^ group (high vs. low, HR: 1.605, Figure 1A). In comparison, survival curves of high and low groups of HER2, a target of the blockbuster antibody drug Herceptin, were between ST14/Prss14^high^ and the ST14/Prss14^low^. When survival curves were examined by separating groups sequentially, first by HER2 levels, and then by ST14/Prss14 levels, differences in the survival profile became clearer. Among the HER2^low^ groups, ST14/Prss14^high^ group showed the poorest survival, while the ST14/Prss14^low^ group showed the greatest survival (HER2^low^/ST14/Prss14^high^ vs. HER2^low^/ST14/Prss14^low^, HR: 4.064, *P* < 0.01, Figure 1B). Among the HER2^high^ groups, the ST14/Prss14 ^low^ group showed poorer survival than the ST14/Prss14^high^ group (HER2^high^/ST14/Prss14^high^ vs. HER2^high^/ST14/Prss14^low^, HR: 0.473).

Next, we wondered whether ST14/Prss14 expression shows any degree of correlation with the stages of TCGA breast cancer used in this analysis (Table 1). The patients in advanced cancer stages III–IV showed poorer survival relative than the patients in the earlier stage I–II (Figure 1C). Although the average of ST14/Prss14 expression did not appear to be significantly different in two stage groups (Figure 1D), the ST14/Prss14^high^ subgroup at stage III–IV showed the poorest survival (III–IV/ST14/Prss14^high^ vs. III–IV/ ST14/Prss14^low^, HR: 1.350, *P* > 0.05, Figure 1E).

**Table 1:** TCGA BRCA patient cohorts by ER expression status and cancer subtypes

	ER status				Breast cancer subtypes				
	whole cohort	negative	positive	un-classified	luminal A	luminal B	triple negative	HER2-enriched	un-classified
Characteristics	*n* = 464	*n* = 109	*n* = 350	*n* = 5	*n* = 190	*n* = 62	*n* = 51	*n* = 22	*n* = 139
**mean age**	58	54	60	55	60	61	52	59	57
**(SD, range)**	(13, 26–90)	(13, 26–82)	(13, 27–90)	(16, 44–80)	(13, 29–89)	(14, 29–88)	(12, 29–82)	(13, 34–80)	(13, 26–90)
**tumor stage**									
I–II (%)	334 (74.7)	82 (75.2)	249 (71.1)	3 (60)	137 (72.1)	45 (72.6)	42 (82.4)	15 (68.2)	95 (68.3)
III–IV (%)	113 (25.3)	23 (21.1)	89 (25.4)	1 (20)	48 (25.3)	16 (25.8)	7 (13.7)	7 (31.8)	35 (25.2)
Unclassified (%)	17 (3.8)	4 (3.7)	12 (3.4)	1 (20)	5 (2.6)	1 (1.6)	2 (3.9)	0 (0)	9 (6.5)

ER status and breast cancer subtypes of 464 TCGA BRCA patients were classified by ER, PR, and HER2 expression determined by the IHC technique. Their tumor stages were derived from the clinical data provided.

We attempted to analyze ST14/Prss14 expression status with known molecular markers that could separate breast cancers into subgroups. Among the three molecular markers, ER, PR and HER2, as shown in Figure 1F, ER status affected the survival (low vs. high, HR: 4.365, *P* < 0.01). Interestingly, the level of ST14/Prss14 expression was significantly higher in the ER^−^ group than in the ER^+^ group (ER^−^ vs. ER^+^, *P* < 0.0001, Figure 1G). Particularly, ST14/Prss14^high^ in ER^−^ showed the worst outcome, while ST14/Prss14^low^ in ER^−^ showed the best (ER^−^/ST14/Prss14^high^ vs. ER^−/^ST14/Prss14^low^, HR: 4.213, *P* < 0.05 Figure 1H). Among the groups of patients divided into four subtypes, triple negative (TN) breast cancer which was negative for these three receptors resulted in the lowest survival rate (Figure 1I) and the expression level of ST14/Prss14 was the highest (TN vs. luminal A, *P* < 0.001, TN vs. luminal B, *P* < 0.0001, TN vs. HER2, *P* > 0.05, Figure 1J).

In order to examine whether the poor survival is due to high ST14/Prss14 expression, and not due to the absence of HER2 in TN, we tried to compare the survival of ST14/Prss14^high^ and ST14/Prss14^low^ populations in the HER2 and TN breast cancer groups. Although the numbers of data in the TCGA breast cancer database are extremely low and all TN patients show high ST14/Prss14 expression, the pattern showed poor survival in ST14/Prss14^high^ patients regardless of HER2 expression (Figure S1A and S1B). From another data set (GSE20685) derived from Taiwanese studies [46], a similar pattern was observed (Figure S1C and S1D). However, in these analyses, statistical significance was weak or not significant, most likely due to the limiting numbers of data points.

Therefore, these results suggested that ST14/Prss14 is an excellent prognosis marker, especially in ER^−^ and TN breast cancers. Moreover, ST14/Prss14 is a highly potential therapeutic target in TN breast cancers when Herceptin is not an option.

### Context dependent ST14/Prss14 expression and function in ER^−^ breast cancer patients

**Figure 2:**
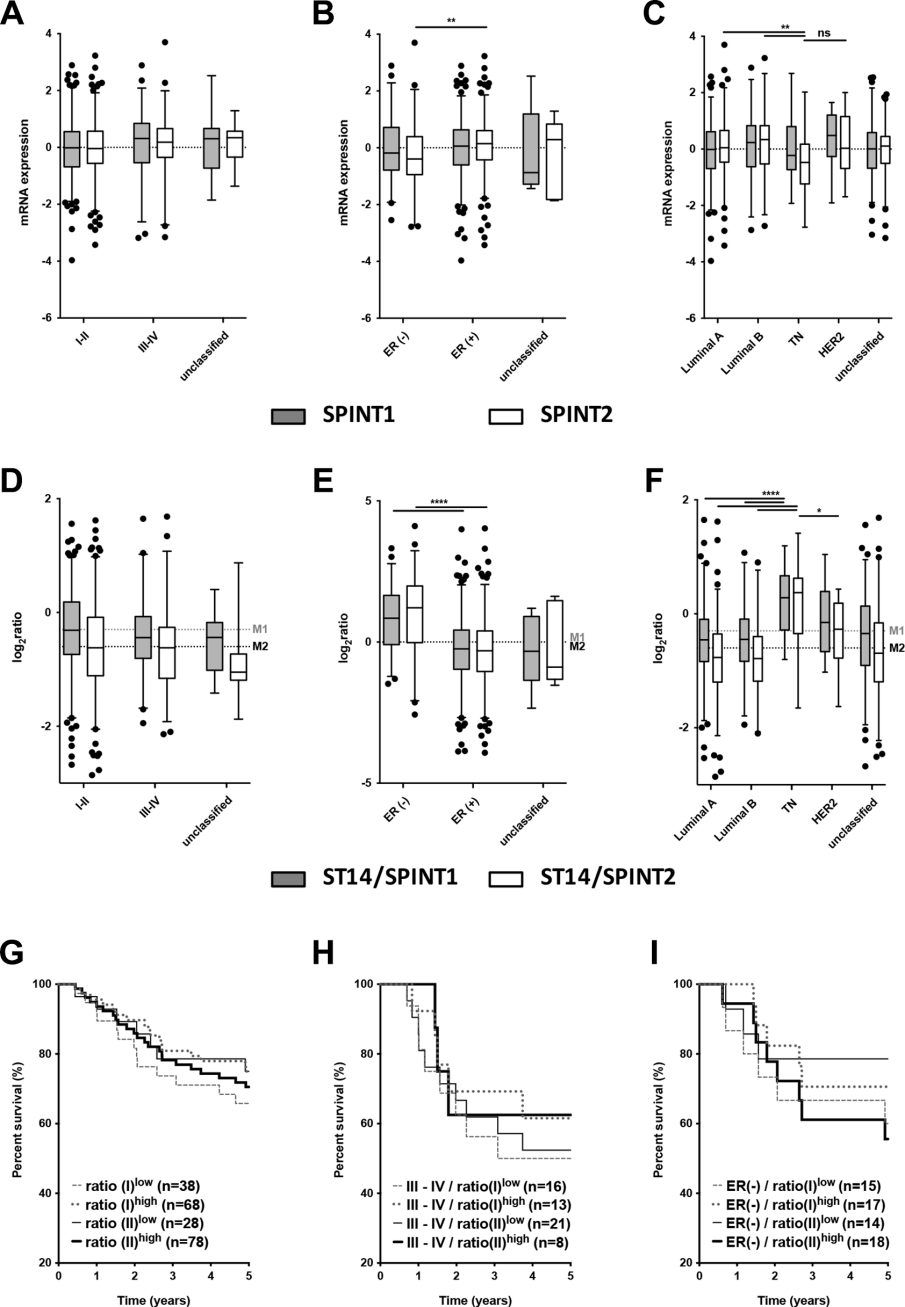
Analysis of the expression of SPINT1, SPINT2 and ratios of ST14/Prss14 to SPINT1 and SPINT2 (**A–C**) Box plots of SPINT1 and SPINT2 expression by cancer stages (I–II, III–IV, and unclassified), by ER expression status (ER^+^, ER^−^, and unclassified), and by breast cancer subtypes (Luminal A, Luminal B, TN, HER2^+^, and unclassified). SPINT1, gray box, SPINT2, white box. (**D–F**) Ratios of ST14/Prss14 to SPINT1 and to SPINT2 by cancer stages (I–II, III–IV, and unclassified), by ER expression status (ER^+^, ER^−^, and unclassified), and by breast cancer subtypes (Luminal A, Luminal B, TN, HER2^+^, and unclassified). ST14/Prss14 to SPINT1, gray box, ST14/Prss14 to SPINT2, white box, average of ST14/Prss14 to SPINT1, M1, average of ST14/Prss14 to SPINT2, M2. (**G–I**) Kaplan-Meier survival curves for the ratios of ST14/Prss14 to SPINT1 (I) and to SPINT2 (II) in stages III–IV group, and ER^−^ group.

Because the increased ratio of ST14/Prss14 to its inhibitor SPINT1 was observed in some carcinomas [36, 37], we examined the ratio of ST14/Prss14 to SPINT1 and SPINT2. While the averages of SPINT1 and SPINT2 expression levels are not different in the cancer stages I–II and III–IV (Figure 2A), SPINT2 expression levels were somewhat lower in ER^−^ and TN breast cancer groups (Mean differences of SPINT2, ER^−^ vs. ER^+^, *P* < 0.01, TN vs. luminal A, *P* < 0.01, TN vs. luminal B, *P* < 0.01, TN vs. HER2. *P* > 0.5) (Figure 2B and 2C). The ratio of ST14/Prss14 to SPINT1, ratio(I), and that of ST14/Prss14 to SPINT2, ratio(II), showed no big difference among the cancer stages (Figure 2D). However, both ratios were significantly higher in ER^−^ (Figure 2E) and TN groups than in others (Figure 2F).

The survival curves examined by these ratios of ST14/Prss14 to inhibitors did not reveal significant differences (Figure 2G). In addition, high and low ratios as well as those in cancer stage III–IV group also did not reveal any significant differences in the survival profiles (Figure 2H). The ratio(II)^high^ is somewhat higher in the ER^−^ group and showed poorer 5 year survival, however, without statistical significance (Figure 2I). These results suggest that inhibitor expression does not critically contribute to patient's survival, particularly for early years. However, larger number of cases can help to conclude whether the protease/inhibitors ratio contribute to the patient's survival.

Table 2 shows a coexpression pattern of known ST14/Prss14 substrates and their receptors/interacting partners. Genes of coexpression profiles with better significance are CUB domain-containing protein 1 (CDCP1), uPA receptor (PLAUR), EGFR, HGF receptor (MET), and desmoglein-2 (DSG2), a component of laminin 5 (LAMB3). These genes are all well-known not only for EMT but also other cancer processes. Among the negatively associated genes, hepsin (HPN), macrophage stimulating protein (MST1), platelet derived growth factor D (PDGFD), and Tie 2 (TEK) showed a stronger correlation. These results suggest that a subset of ST14/Prss14 substrates is involved in breast cancer progression.

**Table 2:** Coexpression of known ST14/Prss14 substrates and their receptors downstream

			Correlation with ST14		ER^−^ vs. ER^+^	
Gene symbol	Alias	Description	Pearson r	*P* value	Mean Diff.	*P* value
ST14	Matriptase, Epithin, PRSS14, MT-SP1	Suppression Of Tumorigenicity 14 (Colon Carcinoma)			0.9469	****
CDCP1	TRASK, CD318	CUB Domain Containing Protein 1	0.4332	****	0.4074	***
PLAUR	uPAR	Plasminogen Activator, Urokinase Receptor	0.3200	****	0.7024	****
EGFR	ERBB1, HER1	Epidermal Growth Factor Receptor	0.2893	****	1.4520	****
MET	HGFR, C-Met	MET Proto-Oncogene, Receptor Tyrosine Kinase	0.2187	****	0.9845	****
DSG2		Desmoglein 2	0.2141	****	0.6692	****
LAMB3		Laminin, Beta 3	0.2064	****	0.4717	****
PLAU	uPA	Plasminogen Activator, Urokinase	0.1719	***	0.1552	ns
PRSS8	prostasin	Protease, Serine, 8	0.1598	***	−0.3147	*
FN1		Fibronectin 1	0.1192	*	−0.0952	ns
MMP3		Matrix Metallopeptidase 3	0.0736	ns	0.2221	ns
MMP2		Matrix Metallopeptidase 2	0.0627	ns	−0.1601	ns
PLG		Plasminogen	0.0405	ns	0.1799	ns
F2RL1	PAR2	Coagulation Factor II (Thrombin) Receptor-Like 1	0.0336	ns	0.0387	ns
KDR	VEGFR2	Kinase Insert Domain Receptor	0.0121	ns	−0.0700	ns
FLG		Filaggrin	−0.0120	ns	−0.0148	ns
KLKB1		Kallikrein B, Plasma (Fletcher Factor) 1	−0.1277	**	−0.1440	ns
HGF		Hepatocyte Growth Factor (Hepapoietin A; Scatter Factor)	−0.1640	***	−0.6772	****
IGFBP7	Angiomodulin	Insulin-Like Growth Factor Binding Protein 7	−0.1783	***	−0.3749	**
TEK	Tie-2	TEK Tyrosine Kinase, Endothelial	−0.1995	****	−0.5076	****
MST1		Macrophage Stimulating Protein	−0.2176	****	−0.6715	****
PDGFD		Platelet Derived Growth Factor D	−0.2249	****	−0.7368	****
HPN	TMPRSS1	Hepsin	−0.2561	****	−1.0260	****

Genes were analyzed for correlation with ST14/Prss14 and ER expression status, using TCGA BRCA data. Genes were arranged by the degree of correlation values with ST14/Prss14.

* *P* ≤ 0.05,

** *P* ≤ 0.01.

*** *P* ≤ 0.001,

**** *P* ≤ 0.0001, ns (not significant).

### Positioning ST14/Prss14 in the cluster of EMT signature genes with breast cancer types

**Figure 3:**
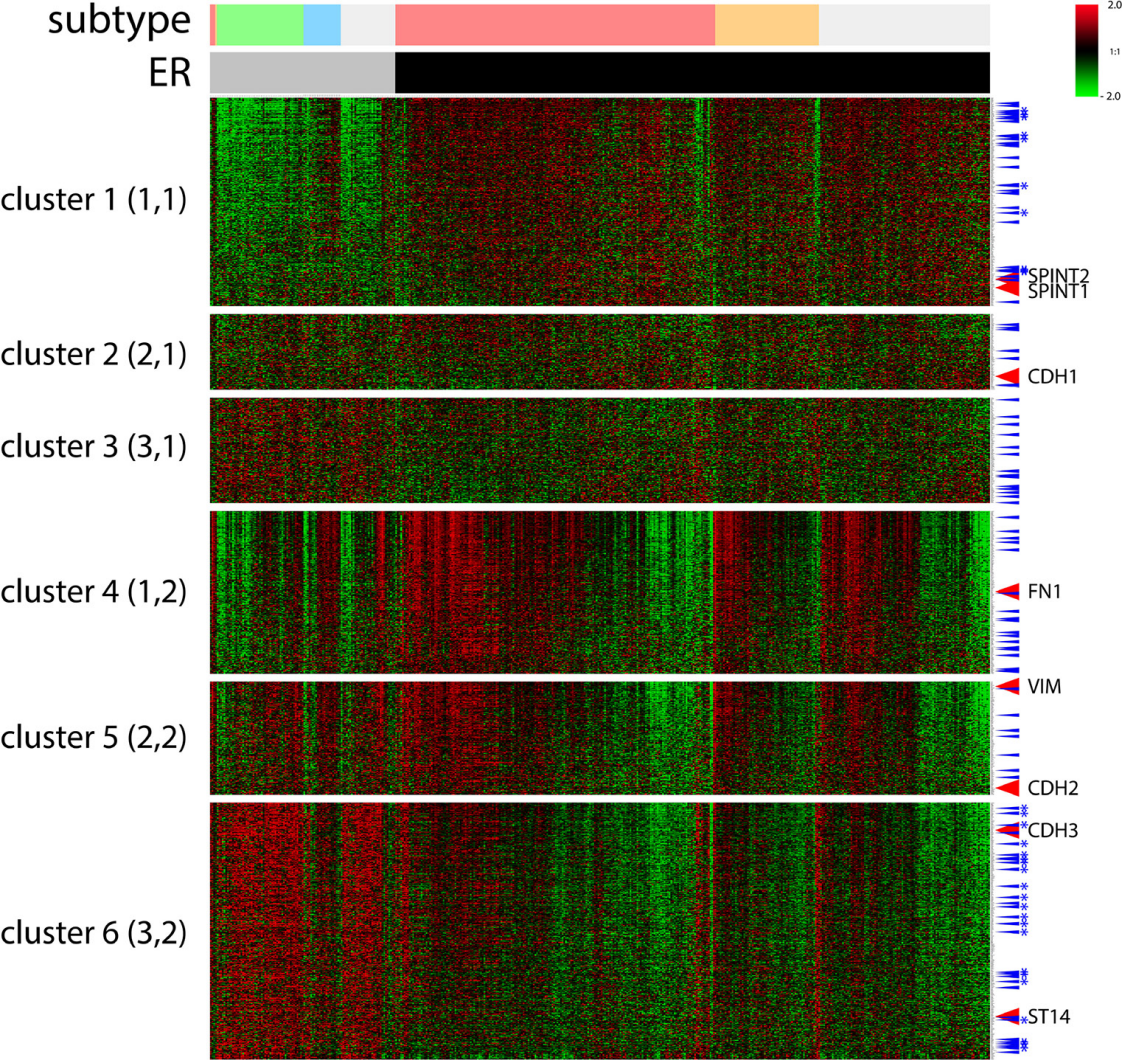
A cluster analysis of 1085 selected EMT signature genes by ER expression status of TCGA BRCA data by a SOM analysis Six clusters (3×2) were applied using an Euclidean distance. ER status: ER^−^, gray bar, ER^+^, black bar. Breast cancer subtypes: luminal A, pink, luminal B, orange, TN, green, HER2, sky blue, unclassified, light gray. TFs, blue arrow, TFs of cluster 1 and 6 common in two different datasets, with stars. Signature EMT markers along with ST14/Prss14 and inhibitors were indicated. Color key: −2.0 (green) to 2.0 (red).

We next positioned ST14/Prss14 in clusters with particular EMT signature genes in breast cancer patients to find clues for the specific role of ST14/Prss14 in the EMT process that we described earlier [14]. Our selection of 1085 EMT signature genes (Table S1) were first clustered according to the cancer stages, using a self-organizing map (SOM) analysis (Figure S2). However, it was difficult to clearly differentiate expression clusters between the two groups of cancer stages (stage I–II or stage III–IV). A clustering pattern did not reveal any satisfying grouping of the known EMT signature genes with the early I–II and the late III–IV stages. Therefore, we used an alternative clustering strategy in which ER expression status of the cancer tissues was taken into consideration (Figure 3). The resulting clusters revealed several differential expressions between the ER^−^ and ER^+^ groups. Cluster 1, of which expression was low in ER^−^ patients, contains the two inhibitors, SPINT1 and SPINT2. On the contrary, cluster 6 containing genes show the opposite pattern to cluster 1 (high in ER^−^, low in ER+ patients). Cluster 6 includes ST14/prss14 and P-cadherin (CDH3). While cluster 2 contains CDH1, cluster 3 does not appear to show a distinctive pattern. In contrast, cluster 4 and cluster 5 contain FN1, VIM, and CDH2. Cluster 4 and 5 showed similar patterns to cluster 6 with minor differences. From these results, the role of ST14/Prss14 during the EMT process cannot be easily resolved since it is not co-clustered with any of the stage specific EMT markers, such as CDH1 or CDH2 (see discussion).

**Figure 4:**
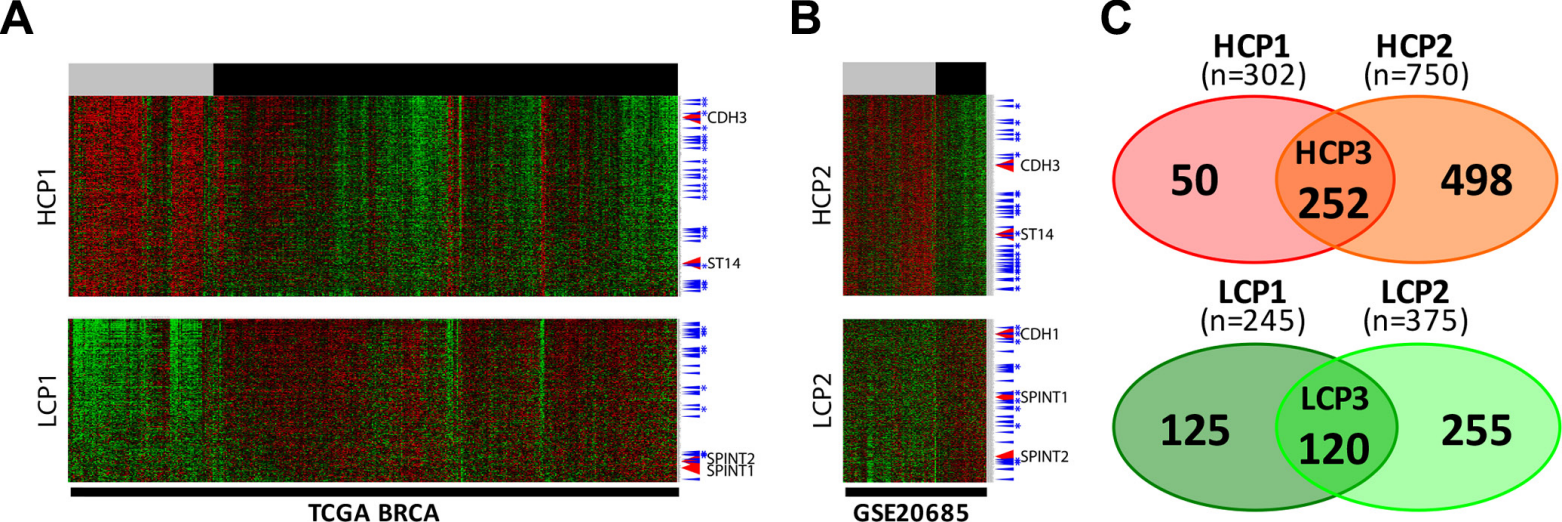
Two EMT signature gene clusters by ER expression status in two different datasets each cluster was selected after the six clusters were divided by a SOM analysis (**A**) TCGA BRCA and (**B**) GSE20685. High expression cluster in the ER^low^ patients group, HCP, low expression cluster in the ER^low^ patients group, LCP. ER status: ER^low^, gray bar, ER^high^, black bar. TFs, blue arrow, TFs common in two different datasets, with stars. Color key: −2.0 (green) to 2.0 (red). (**C**) Two Venn diagram of four gene sets. HCP1 and HCP2 on top, LCP1 and LCP2 at the bottom.

Next, to verify the pattern of TCGA BRCA gene clustering, we chose to analyze another breast cancer dataset from the Gene Expression Omnibus (GEO) database GSE20685. For comparison with the TCGA BRCA dataset, we named the cluster containing genes that are expressed in the higher level in ER^−^ than that in ER^+^ breast cancer cells as Higher Cluster in Patients (HCP). Similarly, for the other cluster containing genes expressed in lower level in ER^−^ breast cancer, we named it as Lower Cluster in Patients (LCP). In the case of the TCGA BRCA dataset, cluster 6 and cluster 1 (Figure 3) are HCP1 and LCP1, respectively. When HCP1 and LCP1 were compared to those of the GEO dataset, HCP2 and LCP2 (Figure 4A–4B), a large proportion of the EMT genes were coclustered (Figure 4C): 252 HCP genes, named as HCP3, are positioned in both HCP1 and HCP2 (Table S2) and 120 LCP genes, named as LCP3, are positioned in both LCP1 and LCP2 (Table S3, Figure 4C).

### Coexpression analyses of EMT signature genes and ST14/Prss14 in ER^−/low^ and ER^+/high^ breast cancer patients

**Figure 5:**
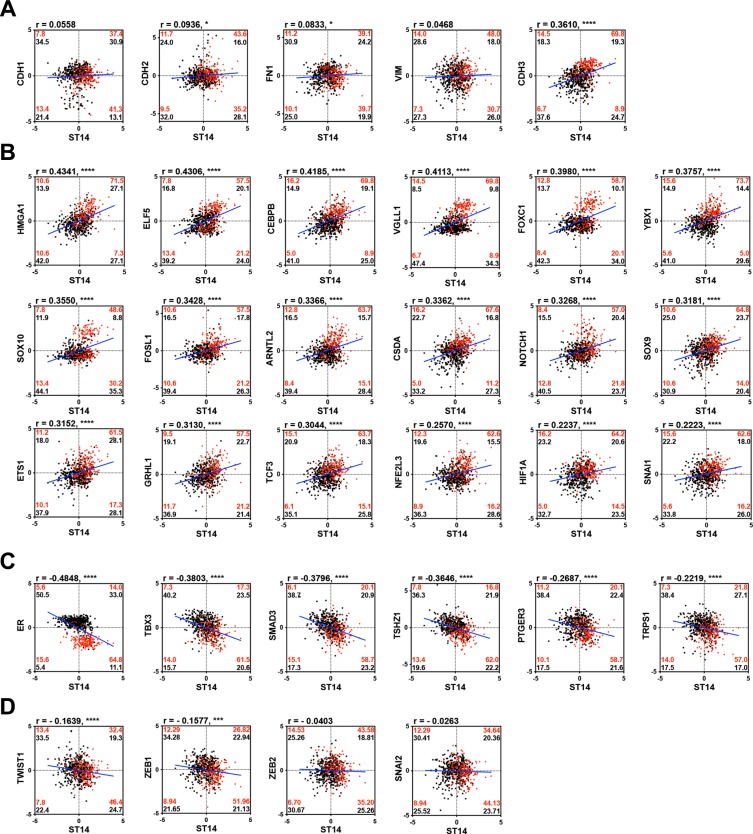
Correlation analysis of well characterized EMT signature genes and TFs with ST14/Prss14 in ER^−/low^ and ER^+/high^ population (**A**) Scatter plots of well-known EMT progression markers, (**B**) common TFs in HCP3, (**C**) LCP3, and (**D**) TFs in other clusters. Correlation coefficient *r* values between two genes were computed using Pearson correlation calculations. Two-tailed *P* values were calculated by an unpaired *t* test. *P < 0.05, ***P < 0.001,****P < 0.0001. Percentages of population by two genes expression in each ER^−/low^ (red) and ER^+/high^ (black) groups were marked in each quadrants. Linear interpolation line in whole patients, blue line.

To study the regulatory mechanism of ST14/Prss14 expression during the EMT process in detail, we next examined the degree of correlation between well-known EMT signature genes and ST14/Prss14 in ER^+/high^ and ER^−/low^ populations (TCGA, *n* = 459, where ER^−^ and ER^+^ was judged by IHC and GSE20685, *n* = 108, where ER^high^ and ER^low^ were determined by above and below the standard deviation based on the average value). Although ST14/Prss14 expression is distinctively high, none of the EMT stage specific genes among CDH1, VIM, FN1, CDH2 showed a clear correlation with ST14/Prss14 (Figure 5A). However, the majority of CDH2 high expressors, low in numbers, reside in the ST14/Prss14 high population. Interestingly relatively less studied P-cadherin (CDH3) showed the significant correlation values both in ER^−/low^ and ER^+/high^ (*P* < 0.001). These analyses suggest that ST14/Prss14 expression in the ER^−/low^ population could not be assigned to any conventional specific EMT stages, pre-EMT or post-EMT states.

Next, coexpression values of well-known EMT TFs in HCP3 and LCP3 clusters, whose protein class was based on PANTHER, are shown in Table 3, where 18 TFs in HCP3 show high positive correlation values (Figure 5B), and 6 TFs in LCP3 show negative correlation values with ST14/Prss14 (Figure 5C). HMGA1, ELF5, CEBPB, and VGLL1 have the highest positive correlation values over 0.4, suggesting probable positive regulation of ST14/Prss14 expression. Interestingly, a majority of YBX1 and HIF1A positive populations in ER^−/low^ breast cancers are also ST14/Prss14 high expressors (Figure 5B). Six TFs (ER, TBX3, SMAD3, TSHZ1, PTGER3, and TRPS1) yielded well distinguishable separate high and low populations, suggesting they may function as negative regulators of ST14/Prss14 in ER^−/low^ populations (Figure 5C).

**Table 3:** Coexpression of common TFs in HCP3 and in LCP3 with ST14/Prss14 among the breast cancer patients

HCP3		Correlation with ST14			LCP3	Correlation with ST14	
Gene symbol	Description	Pearson r	*P* value	Gene symbol	Description	Pearson r	*P* value
HMGA1	High Mobility Group AT-Hook 1	0.4341	****	ESR1	Estrogen Receptor 1	−0.4267	****
ELF5	E74-Like Factor 5 (Ets Domain Transcription Factor)	0.4306	****	TBX3	T-Box 3	−0.3803	****
CEBPB	CCAAT/Enhancer Binding Protein (C/EBP), Beta	0.4185	****	SMAD3	SMAD Family Member 3	−0.3796	****
VGLL1	Vestigial-Like Family Member 1	0.4113	****	TSHZ1	Teashirt Zinc Finger Homeobox 1	−0.3646	****
FOXC1	Forkhead Box C1	0.3980	****	PTGER3	Prostaglandin E Receptor 3 (Subtype EP3)	−0.2687	****
YBX1	Y Box Binding Protein 1	0.3757	****	MSX2	Msh Homeobox 2	−0.2477	****
SOX10	SRY (Sex Determining Region Y)-Box 10	0.3550	****	SPDEF	SAM Pointed Domain Containing ETS Transcription Factor	−0.2394	****
FOSL1	FOS-Like Antigen 1	0.3428	****	TSC22D3	TSC22 Domain Family, Member 3	−0.2328	****
ARNTL2	Aryl Hydrocarbon Receptor Nuclear Translocator-Like 2	0.3366	****	TRPS1	Trichorhinophalangeal Syndrome I	−0.2219	****
CSDA	Y Box Binding Protein 3	0.3362	****	MTA3	Metastasis Associated 1 Family, Member 3	−0.2152	****
NOTCH1	Notch 1	0.3268	****	ZHX2	Zinc Fingers And Homeoboxes 2	−0.1387	***
SOX9	SRY (Sex Determining Region Y)-Box 9	0.3181	****	LEF1	Lymphoid Enhancer-Binding Factor 1	−0.1309	**
ETS1	V-Ets Avian Erythroblastosis Virus E26 Oncogene Homolog 1	0.3152	****	ZBTB38	Zinc Finger And BTB Domain Containing 38	−0.1280	**
GRHL1	Grainyhead-Like 1 (Drosophila)	0.3130	****	GSC	Goosecoid Homeobox	−0.1183	**
TCF3	Transcription Factor 3	0.3044	****	OVOL2	Ovo-Like Zinc Finger 2	−0.0938	*
NFE2L3	Nuclear Factor, Erythroid 2-Like 3	0.2570	****	PBX1	Pre-B-Cell Leukemia Homeobox 1	−0.0580	ns
ZNF532	Zinc Finger Protein 532	0.2538	****	GRHL2	Grainyhead-Like 2 (Drosophila)	−0.0446	ns
ETV5	Ets Variant 5	0.2388	****	RUNX1	Runt-Related Transcription Factor 1	−0.0350	ns
HIF1A	Hypoxia Inducible Factor 1, Alpha Subunit (Basic Helix-Loop-Helix Transcription Factor)	0.2237	****	CREB3L1	CAMP Responsive Element Binding Protein 3-Like 1	0.0558	ns
SNAI1	Snail Family Zinc Finger 1	0.2223	****				
PLAGL1	Pleiomorphic Adenoma Gene-Like 1	0.2187	****				
FOXD1	Forkhead Box D1	0.2177	****				
ZBED2	Zinc Finger, BED-Type Containing 2	0.1272	**				
HMGA2	High Mobility Group AT-Hook 2	0.1219	**				
PRDM1	PR Domain Containing 1, With ZNF Domain	0.1017	*				
ZNF521	Zinc Finger Protein 521	0.0956	*				
PCOLCE2	Procollagen C-Endopeptidase Enhancer 2	0.0688	ns				
MYC	V-Myc Avian Myelocytomatosis Viral Oncogene Homolog	0.0303	ns				

Common TFs in TCGA BRCA and GSE20685 analyzed correlation with ST14/Prss14. TFs in HCP3 and LCP3 were arranged by the degree of correlation values with ST14/Prss14.

* *P* ≤ 0.05,

** *P* ≤ 0.01.

*** *P* ≤ 0.001,

**** *P* ≤ 0.0001, ns (not significant).

In addition, we examined the previously well-characterized EMT TFs outside of HCP3 and LCP3 clusters (Figure 5D). In cases of TWIST1 and ZEB1, they show negative correlations (*r* = −0.1639, *P* < 0.001 and *r* = −0.1577, *P* < 0.01, respectively). ZEB1 is previously shown to be a negative regulator of ST14/Prss14 expression [47]. ZEB2 and SNAI2 did not show strong correlation values with ST14/Prss14 in either ER^−/low^ or ER^+/high^ breast cancers.

Next, we navigated putative cellular function or signal pathways of associated TFs through a text mining tool (Figure S3). The majority of TFs in HCP3 were associated with cell migration, and stem cell development, cancer progression, and metastasis, in addition to EMT. Therefore, TFs which are expressed highly in ER^−/low^ breast cancer populations have a strong connection to cancer progression and metastasis through EMT as well as to regulation of the expressions of ST14/Prss14. In contrast, fewer known functions such as regulation of granulocyte differentiation and steroid hormone receptor activity, were associated with TFs in the LCP3 group.

### Analyses of gene sets with cultured breast cancer cells reveal a paradox in ST14/Prss14 expression

**Figure 6:**
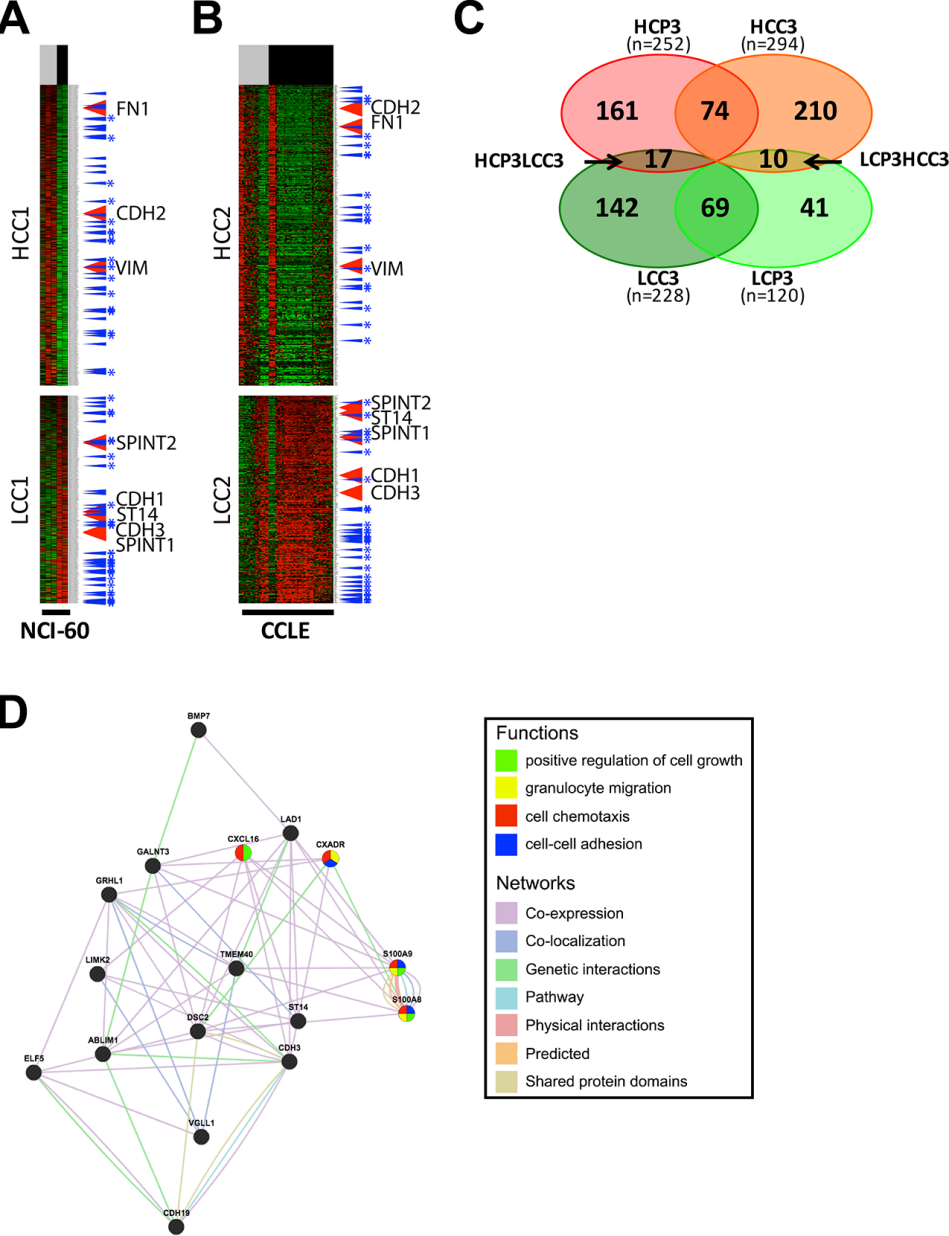
Analysis and gene networking of EMT genes coexpressed in cell lines (**A–B**) EMT signature genes are clustered by ER expression status in breast cancer cell lines of NCI-60 and CCLE. Each cluster was selected after clusters were divided by a SOM analysis. High expression cluster in ER^low^ cell lines group, HCC, low expression cluster in ER^low^ cell lines group, LCC. ER status: ER^low^, gray bar, ER^high^, black bar. TFs, blue arrow, TFs common in two different datasets, blue with stars. (**C**) A Venn diagram of four gene sets, HCP3, LCP3, HCC3, and LCC3. (**D**) Gene network and functional analysis of HCP3LCC3 gene set coexpressed with ST14/Prss14 in HCP3 and LCC3.

In order to further verify the regulatory mechanism of ST14/Prss14 expression, we decided to examine expression of EMT signature genes in cultured breast cancer cell lines. Two sets of mRNA array data of breast cancer cell lines (5 in NCI-60 and 57 in CCLE databases) were sorted according to ER expression status. Next, EMT signature genes were clustered according to the ER status. For comparison with the patients' data (HCPs LCPs), we named the cluster with high expression level in ER^low^ as Higher Cluster in Cell (HCC) and the one with low expression in ER^low^ as Lower Cluster in Cell (LCC). We extracted from two different cell line databases, NCI-60 and CCLE, and named them HCC1/LCC1 (Figure 6A) and HCC2/LCC2 (Figure 6B), respectively. 294 genes (HCC3, Table S4), were identified common in HCC1 and HCC2, while 228 genes (LCC3, Table S5) were identified common in LCC1 and LCC2 (Figure 6C). When HCP3/LCP3 and HCC3/LCC3 were compared, there were only 74 genes in the high group and 69 genes in the low group showing the significant discrepancy between patient data and cell line data. Moreover, some genes were allocated to the opposite side in patient and cell line data. Specifically, 17 genes are members of the HCP3 group in the patient data, but, those of the LCC3 group in the cell line data (Figure 6C). ST14/Prss14 is one of such genes: its expression was low in ER^low^ breast cancer cell lines, clustered together with CDH1 and its two inhibitors, SPINT1 and SPINT2 (Figure 6A and 6B). We also identified 10 genes differentially positioned from LCP3 to HCC3 (Figure 6C). The list of genes that switched clustering positions from the high to the low (HCP3LCC3) and from the low to the high (LCP3HCC3) are listed in Table 4.

**Table 4:** List of HCP3LCC3 and LCP3HCC3 genes

HCP3LCC3		LCP3HCC3	
Gene symbol	Description	Gene symbol	Description
ABLIM1	Actin Binding LIM Protein 1	FGFR1	Fibroblast Growth Factor Receptor 1
BMP7	Bone Morphogenetic Protein 7	GALNT5	Polypeptide N-Acetylgalactosaminyltransferase 5
CDH19	Cadherin 19, Type 2	IL6ST	Interleukin 6 Signal Transducer
CDH3	Cadherin 3, Type 1, P-Cadherin (Placental)	NAV3	Neuron Navigator 3
CXADR	Coxsackie Virus And Adenovirus Receptor	PHLDB2	Pleckstrin Homology-Like Domain, Family B, Member 2
CXCL16	Chemokine (C-X-C Motif) Ligand 16	POLK	Polymerase (DNA Directed) Kappa
DSC2	Desmocollin 2	SLC4A7	Solute Carrier Family 4, Sodium Bicarbonate Cotransporter, Member 7
ELF5^†^	E74-Like Factor 5 (Ets Domain Transcription Factor)	SMAD3^†^	SMAD Family Member 3
GALNT3	Polypeptide N-Acetylgalactosaminyltransferase 3	THBS1	Thrombospondin 1
GRHL1^†^	Grainyhead-Like 1 (Drosophila)	ZBTB38^†^	Zinc Finger And BTB Domain Containing 38
LAD1	Ladinin 1		
LIMK2	LIM Domain Kinase 2		
S100A8	S100 Calcium Binding Protein A8		
S100A9	S100 Calcium Binding Protein A9		
ST14	Suppression Of Tumorigenicity 14 (Colon Carcinoma)		
TMEM40	Transmembrane Protein 40		
VGLL1^†^	Vestigial-Like Family Member 1		

Genes coexpressed in breast cancer patients and cell lines were analyzed for coexpression with ST14/Prss14. The list was arranged by the degree of correlation values with ST14/Prss14.

† Transcription factor.

A gene networking of the 17 genes in HCP3LCC3 were searched for putative functional pathways through text mining (Figure 6D). Most genes have genetic interaction among them or the proteins they encode are co-localized. In their subcellular localization according to Genecard (http://www.genecards.org), the majority of gene products are present in the extracellular space, the plasma membrane, and/or the nucleus (Table 5). Some of the genes were apparently associated with cell growth, granulocyte migration, cell chemotaxis, and cell-cell adhesion. Many genes are localized in the extracellular space and/or plasma membrane, suggesting participation in the progression of ER^−^ breast cancer responding to external stimulus.

**Table 5:** Subcellular localization of genes in the HCP3LCC3 gene set

Gene symbol	Description	Protein Class	Extracellular space	Plasma membrane	Cytosol	Cytoskeleton	Golgi apparatus	Mitochondrion	Peroxisome	Lysosome	Endoplasmic reticulum	Nucleus
ST14	Suppression Of Tumorigenicity 14 (Colon Carcinoma)	enzyme	o	o								
GALNT3	Polypeptide N-Acetylgalactosaminyltransferase 3	enzyme	o	o			o	o	o	o	o	
LIMK2	LIM Domain Kinase 2	enzyme			o	o						o
CXADR	Coxsackie Virus And Adenovirus Receptor	cell adhesion	o	o							o	o
DSC2	Desmocollin 2	cell adhesion	o	o		o					o	
CDH3	Cadherin 3, Type 1, P-Cadherin (Placental)	cell adhesion	o	o	o		o		o	o	o	
CDH19	Cadherin 19, Type 2	cell adhesion		o					o			
LAD1	Ladinin 1	structural protein	o		o	o						o
ABLIM1	Actin Binding LIM Protein 1	cytoskeletal protein			o	o						o
S100A9	S100 Calcium Binding Protein A9	signaling molecule	o	o	o	o						o
S100A8	S100 Calcium Binding Protein A8	signaling molecule	o	o	o	o		o				o
CXCL16	Chemokine (C-X-C Motif) Ligand 16	chemokine	o	o								o
BMP7	Bone Morphogenetic Protein 7	growth factor	o	o	o	o	o	o	o	o		o
GRHL1	Grainyhead-Like 1 (Drosophila)	transcription factor	o	o	o		o	o	o		o	o
VGLL1	Vestigial-Like Family Member 1	transcription factor	o									o
ELF5	E74-Like Factor 5 (Ets Domain Transcription Factor)	transcription factor	o		o				o			o
TMEM40	Transmembrane Protein 40	not known							o			

Location of expressed proteins in the HCP3LCC3 gene list was marked in gray. Through the COMPARTMENTS database (http://compartments.jensenlab.org/Search), all cases which were reported more than one were shown.

**Figure 7:**
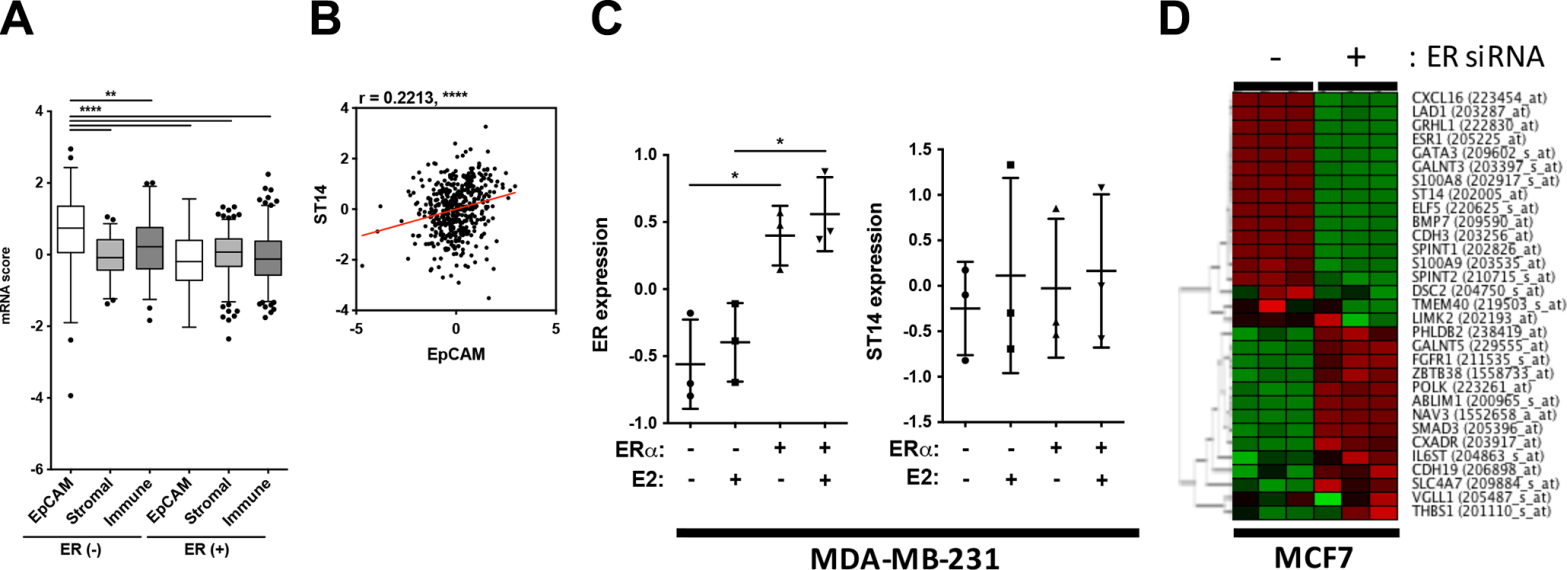
Evaluation of ST14/Prss14 expression in breast cancer patients and cancer cell lines (**A**) ST14/Prss14 expression in cancer tissue or cancer microenvironment using signature gene analysis. A box plot of mRNA scores of EpCAM, stromal signature, and immune signatures separated by ER expression status. (**B**) A scatter plot of correlation between ST14/Prss14 and EpCAM. Linear interpolation curve between two genes in TCGA BRCA patients - red line. *r* = 0.2213, *P* < 0.0001. (**C**) ER and ST14/Prss14 expression with ER gene introduction (ERα) and 17 beta-estradiol (E2) treatment in MDA-MB-231 cell. The line in the middle is plotted at the point of means. *P < 0.05. (**D**) A cluster of HCP3LCC3 and LCP3HCC3 gene sets by ER expression in MCF7 cells. Cluster was examined by HCL analysis, using Euclidean distance. Color key: −1.95 (green) to 1.95 (red).

Due to these apparent discrepancies of gene expression patterns between patient and cell line data, we decided to look at these outliers, HCP3LCC3 and LCP3HCC3, with more attention. To exclude the possibility that the increased expression level of ST14/Prss14 in ER^−^ patients may have resulted from other types of cells in the cancer microenvironment, we analyzed stromal or immune signature genes (Figure 7A). The expressions of stromal and immune signature genes did not show any bias, but were generally similar in ER^−^ and ER^+^cancers. However, the epithelial cell specific adhesion molecule (EpCAM) is distinctively higher in the ER^−^ population than in the ER^+^ population (Figure 7A), showing strong positive correlation with ST14/Prss14 (*r* = 0.2213, *P* < 0.001) (Figure 7B). These results suggest that high expression of ST14/Prss14 in the ER^−^ population is more likely coming from the cancer tissue rather than from the stromal or immune cells.

In an attempt to elucidate the underlining mechanism of regulating ST14/Prss14 expression, we searched the GEO database to get a hint of regulation by exogenous signals, for example, estrogen that is provided from noncancer cells. A basal type ER^−^ MDA-MB-231 cell shows undetectably low ST14/Prss14 expression in western blotting (data not shown). When ER is introduced into cell by transfection, ST14/Prss14 expression does not change either by estrogen induction and/or the presence of ER (Figure 7C). In the case of MCF7, a luminal A type ER^+^ ST14/Prss14 expressing breast cancer cell line, ST14/Prss14 expression was diminished when ER expression was depleted by siRNA (Figure 7D). The other 12 out of 16 genes showing the coexpression profile also behaved similarly in the cluster analysis with the conditions by removing ER expression. Furthermore the genes negatively correlated in their expression behaved in an opposite fashion. These results suggest that ST14/Prss14 expression depends on both ER and the cellular environments in ER^+^ cell lines. Therefore, regulation of ST14/Prss14 is independently regulated by different mechanisms in ER^+^ and ER^−^ breast cancer cell lines.

## DISCUSSION

In this report, we evaluated ST14/Prss14 for a prognosis marker and a therapeutic target to ER^−^ breast cancer by utilizing various public data.

### ST14/Prss14 alone is sufficient to be an independent prognosis marker for ER^−^ breast cancer

Survival of breast cancer patients with higher levels of ST14/Prss14 expression is poor for all breast cancer populations regardless of cancer stages (Figure 1A and 1E). The value of ST14/Prss14 as a prognosis marker is even higher than that of the well known therapeutic target HER2 (Figure 1A). For breast cancer patients with little HER2 expression, ST14/Prss14 expression is especially useful as a prognosis marker (Figure 1B), and therefore for a therapeutic target. ER expression is particularly important to draw a correct prognosis. Expressions of ER and ST14/Prss14 in TCGA and GEO breast cancer patients are clearly in separate populations (Figure 5C). In ER^−^ patients, all the low ST14/Prss14 expressors survived while half of the high expressors survived less than 5 years (Figure 1H). Therefore, an expression analysis of ST14/Prss14, for example by classical immunohistochemical analysis as performed in earlier reports [48, 49], together with those of ER and HER2 would provide an added benefit to predict the prognosis of breast cancer patients.

The ratios of ST14/Prss14 to its inhibitors showed minor correlative difference with survival rates of breast cancer patients (Figure 2). The same was true for the survival rates of the cancer stage III–IV group or the ER^−^ group. These results indicate the ST14/Prss14 expression itself, but not the ratios to the inhibitors, is critical for the prognosis as proposed earlier in some other types of cancer. The contradiction to earlier reports [36, 37] showing the importance of the ratio in malignant tumor progression can be due to smaller sample sizes and, more likely, lack of genetic classification of breast cancer subtypes in those studies.

Network analysis of expression profiles of ST14/Prss14 substrates and its interactors revealed a context dependent behavior in breast cancer (Table 2). A probable pathway in breast cancer included CDCP1 that has known function in cell migration and cell matrix attachment [50]. Other genes positively associated with ST14/Prss14 include PLAUR, EGFR, and MET. These genes are also well documented for participation in epithelial cancer progression. HPN, PDGFD, MST1 are negatively associated genes, although, in weaker strength. The positively associated genes can play intrinsic roles in epithelial cancer progression together with ST14/Prss14, while the negatively associated genes such as PDGFD and MST1, since they are secreted proteins, may also cooperate with ST14/Prss14 for affecting cancer progression when released from stromal and immune cells near cancer. Because ST14/Prss14 can be activated by an autoactivation mechanism, it may work as a master key activating these substrates and thus initiating various signaling pathways on cancer cells (and/or stromal and immune cells nearby) during ER^−^ breast cancer progression and metastasis.

### Does ST14/Prss14 play an alternative role in EMT pathways?

In a previous experiment with various epithelial cell lines, we showed ST14/Prss14 is necessary and sufficient for the EMT process [14]. In the other hand, SPINT1 was also previously shown to be involved in the EMT process [51]. However, the detailed mechanisms of how they participate in EMT is still unknown. In fact, our understanding for the EMT process is very limited although the stages can be divided into 4 steps [43]. The EMT process may also be comprised of multiple pathways as well as multiple steps, and not likely just turning on and off genetic switches.

Our initial search for the clues of ST14/Prss14′s roles in the EMT process was done by clustering a total of 1085 selected EMT signature genes (Table S1) from TCGA and GEO breast cancer patient data (Figure S2 and Figures 3, 4). Those EMT signature genes in the breast cancer patients, when grouped according to ER expression level first, were clustered into 6 groups with distinct patterns (Figure 3). Typical EMT stage specific markers are positioned in different clusters showing differential expression patterns based on breast cancer subtypes. The position of ST14/Prss14 in the cluster is near post EMT markers, suggesting it may function in post EMT stages. However, a lack of a strong correlation of ST14/Prss14 with any of stage specific markers suggests ST14/Prss14 behaves in an unorthodox way.

Next, the same set of selected EMT signature genes were applied for the same analyses with the datasets from breast cancer cell lines (NCI-60 and CCLE databases, Figure 6). Interestingly, ST14/Prss14 expression was positioned in closer association with CDH1 expression in clustering. These results are consistent with previous reports on various types of cancer cell lines [52, 53]. This is in contrast to the fact that the position of ST14/Prss14 is closer to CDH2 in our patient's data revealing a possible discrepancy in the data between patient and cell line (see discussion later).

To understand the regulation of gene expression, coexpressing TFs were searched. Among the well known key TFs in EMT, SNAI1 in HCP3, TWIST 1 and ZEB1 appear on the list for coexpressors (Figure 5B and 5D). When degrees of correlations between expression levels of ST14 and TFs were calculated in ER^−^ populations as shown in Figure 5B, HMGA1, ELF5, CEBPB, VGLL1, showed higher correlation (*r* > 0.4) in the ER^−^ population. A strongly associated gene, HMGA1 is still a mystery regarding its role in EMT. When those genes are networked by functions in Figure S3A, a majority of the genes are linked but with still uncharacterized functions. The most prominent features of the output, so far, include cell migration and Notch signaling in addition to EMT function. From these results altogether, we concluded that ST14/Prss14 is regulated in yet to be determined steps and pathways during the EMT process. Experimental approaches from these results will provide further details.

ST14/Prss14 is located in the clusters of ER^−^ breast cancer patients but in the ER^+^ cell lines. As seen in Figures 3 and 5C, all ER^−^ cancers show high ST14/Prss14 expression. However, ST14/Prss14 as well as CDH3 shifted their location to the low group (LCC3) in cell lines from the high group (HCP3) in patients (HCP3LCC3). In addition to ST14/Prss14, other genes in HCP3LCC3 are ABLIM1, BMP7, CDH19, CDH3, CXADR, CXCL16, DSC2, ELF5, GALNT1, GRHL1, LAD1, LIMK2, S100A8, S100A9, TME40, VGLL1 (Table 4 and Table 5). Three transcription factors, GRHL1, VGLL1, and ELF5 are strong candidates for the cell autonomous regulation of ST14/Prss14 in basal type ER^−^ breast cancer patients and ER^+^ luminal A type breast cancer cell lines.

### Paradox of ST14/Prss14 expression in breast cancer patient and in cell lines regarding ER expression and cell subtypes

In search of explanations of this apparent paradox of ER expression and ST14/prss14 in breast cancer patients and cell lines, we approached the issues as follows. Since ST14/Prss14 is able to shed into the media [7, 11–14, 54], it may therefore have function in trans. Therefore, ST14/Prss14 expression can be also contributed from a cancer microenvironment. Our earlier finding on the expression in activated macrophage [16] also supports this idea. However, correlation analysis of ST14/Prss14 and the specific epithelial maker EpCAM showed statistically significant values for positivity while stromal/immune signature genes show no correlation (Figure 7B). We interpret these as supporting results that the majority of the ST14/Prss14 message is coming from the ER^−^ cancer tissue. In addition, a study involving IHC shows ST14/Prss14 expression in cancer tissue, not in stromal tissue [55]. However, we cannot formally exclude that part of ST14/Prss14 expression is coming from immune cells such as from activated macrophages. To resolve this issue, more careful experimental approaches are necessary.

The resolution of this paradox appears not so simple at this point. As seen in the coexpression analysis of known substrates (Table 2), only part of known ST14/Prss14 functions are likely to be active in a breast cancer context. Therefore a context dependent role of this protein may also take part in eliciting variables, including often contradictory roles as is documented in the case of BMP7 [56, 57]. It may also be dependent on the three dimensional tissue structure and/or to particular extracellular matrix proteins as suggested in the networking of integrins [58]. Any of the above proposals cannot completely explain the complicated paradox at this point and answers to questions will need to wait for many more experiments.

### An important issue in biomarker and therapeutic target discovery using cell line models

In any case, detail mode of action studies on the mechanism of ST14/Prss14 expression in different cell populations, cancer tissues, and the microenvironment will be necessary to target ST14/Prss14 with therapeutic approaches. This paradoxical gene expression profiles serious perils if prognosis and therapeutic evaluations are derived solely from the cell cultures. As seen in an earlier analysis with cell lines and patients' data [59], smaller scales without subgrouping breast cancer types may lead to the contradicting results [59]. Although expression profiling of the breast cell lines was claimed to provide proper models for breast cancers [60], it is critical to include whole gene profiling with patients cancer samples, not only for marker discovery and correct prognosis, but also for individual based therapeutics in the future.

## MATERIALS AND METHODS

### Datasets

Two public datasets for breast cancer patients were obtained from the Cancer Genome Atlas Project (TCGA) (http://cancergenome.nih.gov/) and NCBI Gene Expression Omnibus (GEO) (http://www.ncbi.nlm.nih.gov/geo/). The gene expression data include breast invasive carcinoma (BRCA) patients (level 3), enrolled from 2010–2013. The original 603 patients' data were reduced to 464 patients after removing duplicated samples. The only data annotated with the clinical data were selected for use in the analysis. Two array expression datasets of breast cancer cell lines were derived from the CellMiner database (http://discover.nci.nih.gov/cellminer/home.do) and the Cancer Cell Line Encyclopedia (CCLE, http://www.broadinstitute.org/ccle/home). Agilent mRNA array data of NCI-60 cell lines in the CellMiner database provided 5 breast cancer cell lines. Normalized mRNA expression data of CCLE had 57 breast cancer cell lines chosen from about 1,000 different cancer cell lines. Two experiment sets of data, regulating the expression of ER in MCF7 and MDA-MB-231, were obtained from GEO (GSE27473 and GSE2251).

### Group classification

TCGA patients were classified by cancer stages, I–II and III–IV based on the report with clinical data. Four breast cancer subtypes, luminal A, luminal B, triple negative, and HER2-enriched, were classified by the IHC status of ER, PR, and HER2. After dividing into luminal and non-luminal types by expression of ER and/or PR, four subtypes were determined according to HER2 expression. ER high/low groups of GSE20685 were differentiated by mean ± standard deviation of ER. Breast cancer cell lines of NCI-60 and CCLE were grouped by only the mean of ER because of limited sample sizes. Gene high/low groups were determined by mean of each gene in all normalized samples of each dataset. In case of grouping by ER expression status, only TCGA data were divided into negative (−) or positive (+).

### Genesets

EMT signature genes were collected from three commercial data (EMT PCR arrays, QIAGEN/EMT antibody sampler kit, #9782, Cell Signaling technology/cell biology research, R&D), two patents (US20120302572 A1 and US20140155397 A1), and nine studies [49, 51, 61–67]. Genes in the downstream pathway of ST14 were taken from previous publications [15, 19, 21, 24, 31, 68–78]. All gene sets were divided by the standard deviation of each gene after mean centering genes for gene normalization, using software Genesis (http://genome.tugraz.at).

### Statistical analysis

For the 5 year survival curves, data of TCGA BRCA patients were processed in a Kaplan-Meier survival analysis after excluding those whose contacts were lost. The *P* value was calculated using a Log-rank (Mantel-Cox) test and the hazard ratio (HR) was determined by the Mantel-Haenszel method (http://www.graphpad.com/guides/prism/6/statistics/index.htm?stat_the_hazard_ratio.htm). For the mRNA expression analysis, normalized values were drawn in box plots. The differences between multiple groups were assessed by a one-way ANOVA. A ratio of ST14/Prss14 to SPINT1 and SPINT2 was calculated by substituting values with the logarithm to the base of 2. For the correlation analysis, expression values of all patients were drawn in scatter plots with linear interpolation curves between the two genes. The correlation coefficient *r* values between the two genes were computed using Pearson correlation calculations. Two-tailed *P* values were calculated by an unpaired *T* test. The populations of TFs and ST14 by ER status were computed into percentages against each ER^low^ and ER^high^. To analyze stromal and immune scores in the TCGA BRCA dataset, those signature genes, referred to in the publication [79], were selected. After normalization, each stromal and immune score was calculated as an average. All statistical analyses were performed in GraphPad Prism (version 6).

### Cluster analysis

Cluster analyses were examined with self-organizing maps (SOM) using Euclidean distances and calculated 2 to 6 clusters [80]. It was iterated 100,000 times.

### Functional analysis

Gene networking was analyzed by GeneMANIA (http://www.genemania.org/). Networks between genes such as co-expression, co-localization, genetic interaction, pathway, physical interactions, predicted, and shared protein domains were indicated with different colored lines. The results of co-expression were referenced by the top 10% publication size (29/287). Network weighting was based on molecular function of Gene Ontology. The functions of genes, which had a low value, were additionally marked with colors. Subcellular localizations of the gene set were obtained from the COMPARTMENTS database (http://compartments.jensenlab.org/Search). Protein classes of those genes were classified based on PANTHER (http://pantherdb.org/).

## SUPPLEMENTARY MATERIALS FIGURES AND TABLES












