# Supplementary material for: Impact of suppression of tumorigenicity 14 (ST14)/serine protease 14 (Prss14) expression analysis on the prognosis and management of estrogen receptor negative breast cancer
Source: Oncotarget. 2016 May 4;7(23):34643–63. doi: 10.18632/oncotarget.9155 (PMC5085182; doi:10.18632/oncotarget.9155)
Supplement: Supplementary file 1 [file oncotarget-07-34643-s001.pdf]

Impact of suppression of tumorigenicity 14 (ST14)/serine protease 14 (Prss14) expression analysis on the prognosis and management of estrogen receptor negative breast cancer

Supplementary Materials

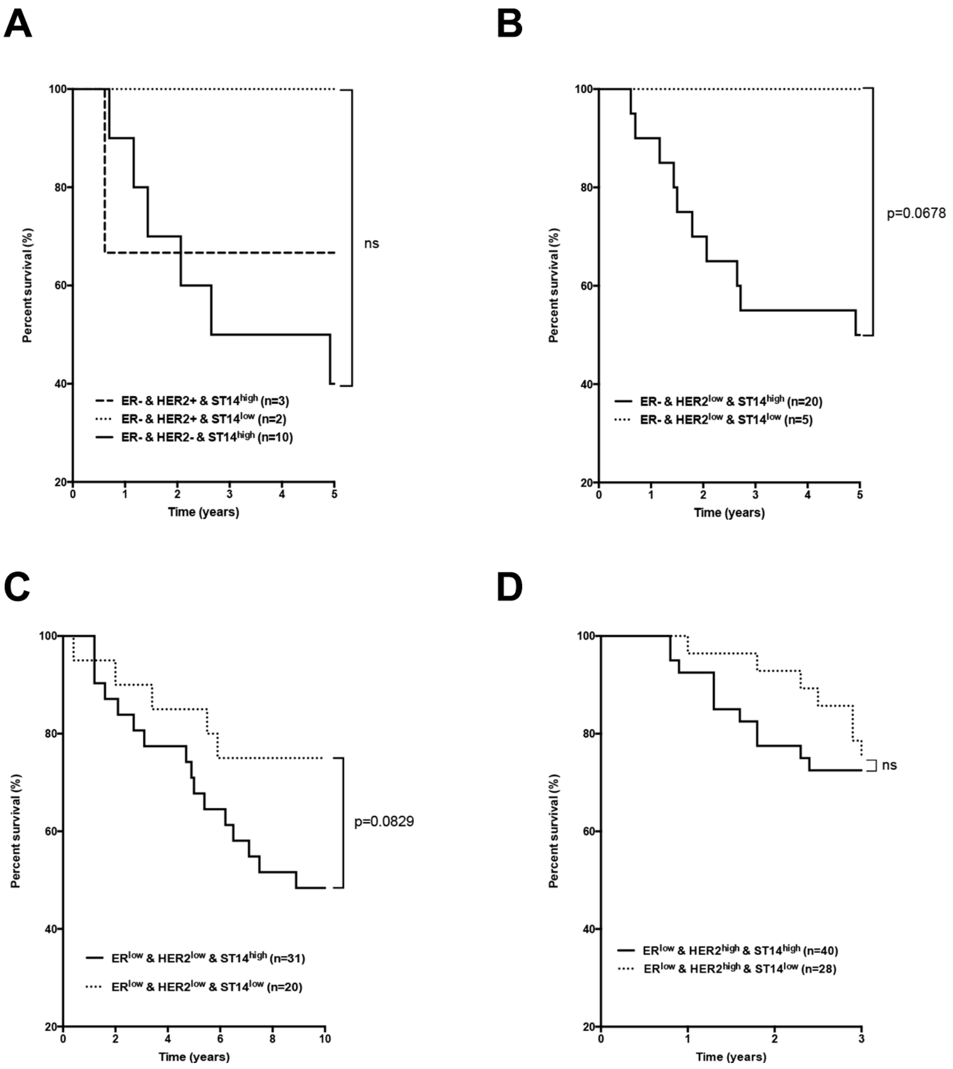

Supplementary Figure S1: Survival curves of ER$^{-/low}$ patients by HER2 and ST14 expression levels. (**A**) Kaplan-Meier survival curves for three groups in TCGA dataset. ER and HER2 status were determined by IHC. ST14/Prss14 high or low are determined based on Array data. (**B**) Kaplan-Meier survival curves for ST14/Prss14 high or low in ER- & HER2^{low} patients in TCGA dataset. ER status is determined by IHC, and HER2 status is determined by relative expression levels. (**C**) Kaplan-Meier survival curves for ST14/Prss14 high or low in ER^{low} and HER2^{low} patients in GSE20685 dataset. (**D**) Kaplan-Meier survival curves for ST14/Prss14 high or low in ER^{low} and HER2^{high} patients in GSE20685 dataset. Both ER and HER2 status are determined by relative expression levels. *P* values were calculated by Log-rank (Mantel-Cox) test.

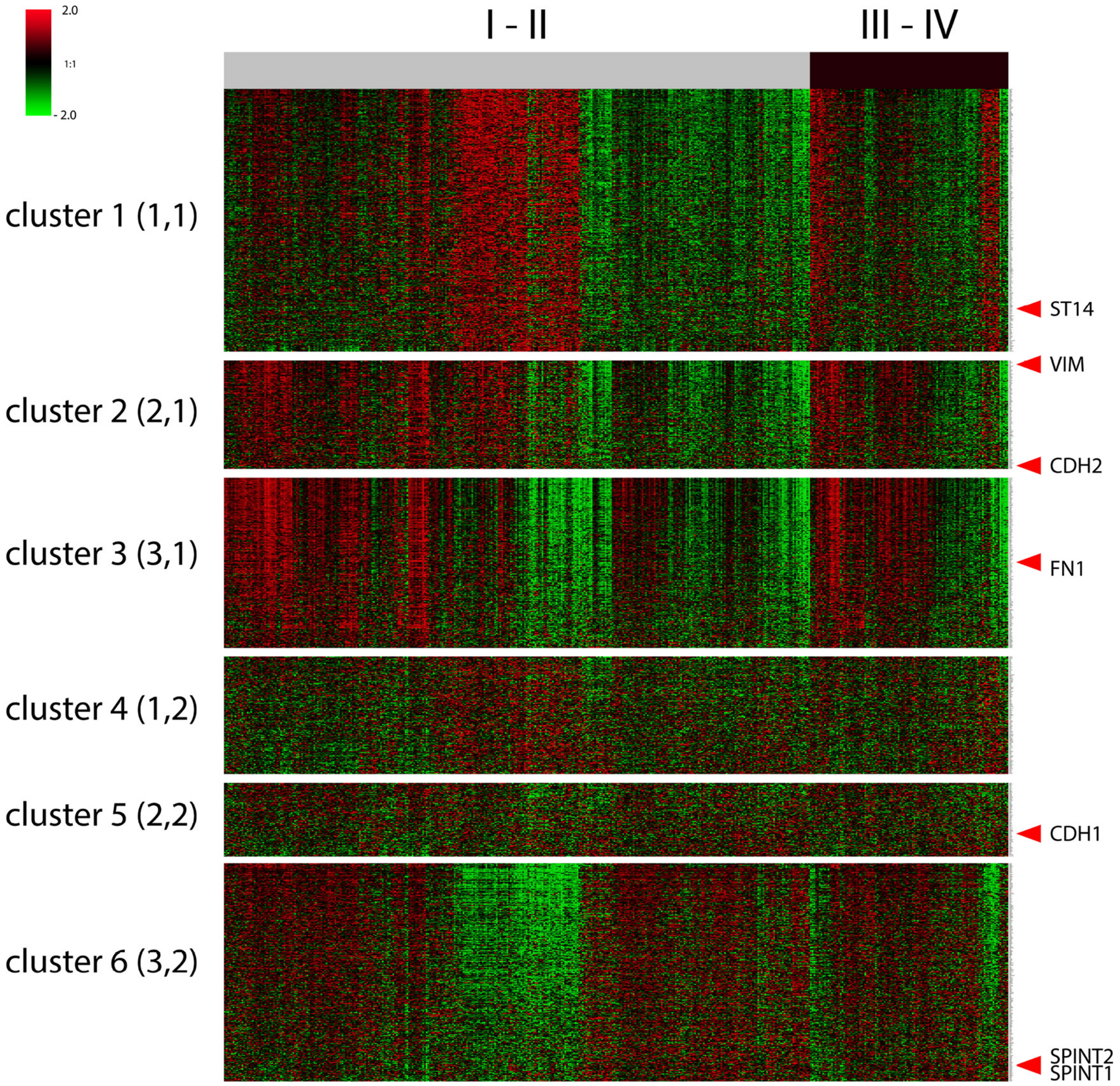

Supplementary Figure S2: A cluster analysis of EMT signature genes by cancer stages of TCGA BRCA database. ST14/Prss14 inhibitors SPINT1, SPINT2, 4 stage specific EMT signature genes, CDH1, VIM, FN1, CDH2 and well known TFs are shown on the right. Six clusters (3 × 2) were divided by SOM analysis, using Euclidean distance with 100,000 iteration. Cancer stages: I–II, gray bar, III–IV, black bar. Color key: –2.0 (green) to 2.0 (red).

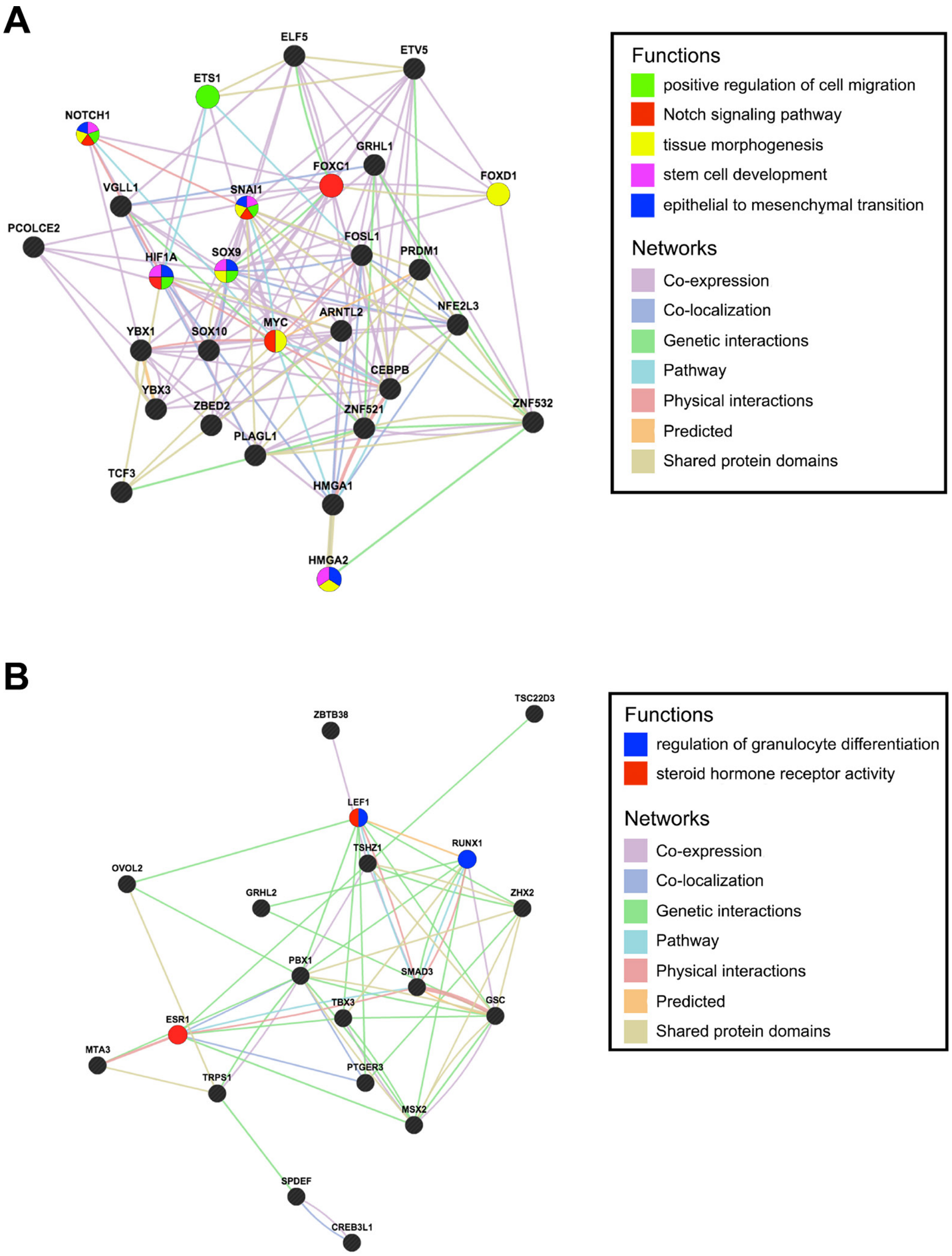

Supplementary Figure S3: Diagram of networking cellular function of TFs. TFs in (**A**) HCP3 and in (**B**) LCP3. Color coded functions and networks are described in the boxes. Unknown functions are in black circles. Lines indicate connections.

Supplementary Table S1: 1085 EMT signature genes list. See Supplementary_Table_S1

Supplementary Table S2: HCP3 genes list. See Supplementary_Table_S2

Supplementary Table S3: LCP3 genes list. See Supplementary_Table_S3

Supplementary Table S4: HCC3 genes list. See Supplementary_Table_S4

Supplementary Table S5: LCC3 genes list. See Supplementary_Table_S5
